# P_2_Y_12_ receptor blockers are anti-inflammatory drugs inhibiting both circulating monocytes and macrophages including THP-1 cells

**DOI:** 10.1038/s41598-021-95710-3

**Published:** 2021-08-31

**Authors:** Patrick M. Siegel, Laura Sander, Alba Fricke, Johannes Stamm, Xiaowei Wang, Prerna Sharma, Nicole Bassler, Ya-Lan Ying, Christoph B. Olivier, Steffen U. Eisenhardt, Christoph Bode, Ingo Ahrens, Philipp Diehl, Karlheinz Peter

**Affiliations:** 1 Department of Cardiology and Angiology I, Faculty of Medicine, University Heart Center Freiburg - Bad Krozingen, University of Freiburg, Freiburg, Germany; 2 Atherothrombosis and Vascular Biology Laboratory, Baker Heart and Diabetes Institute, 75 Commercial Road, Melbourne, VIC 3004 Australia; 3 Department of Medicine, Central Clinical School, Monash University, Melbourne, Australia; 4 Baker Department of Cardiometabolic Health, University of Melbourne, Melbourne, Australia; 5 Department of Plastic and Hand Surgery, Faculty of Medicine, University of Freiburg, Freiburg, Germany; 6 Department of Cardiology and Medical Intensive Care, Augustinerinnen Hospital, Cologne, Germany

**Keywords:** Cardiovascular biology, Translational research, Cardiology, Medical research

## Abstract

P_2_Y_12_ blockade improves patient outcomes after myocardial infarction. As well as antithrombotic effects, anti-inflammatory effects may contribute to this beneficial clinical outcome. Here we aimed to identify potential anti-inflammatory effects of P_2_Y_12_ receptor blockers on monocytes and macrophages. Using flow cytometry, migration assays, flow chambers and RNA microarrays, we investigated the effects of adenosine diphosphate (ADP) and P_2_Y_12_ receptor blockers on blood monocytes, THP-1 monocytes and THP-1 monocytes after differentiation to macrophages. P_2_Y_12_ -expressing platelets can form aggregates with monocytes in circulating blood. Mediated by platelets, ADP results in activation of the integrin receptor Mac-1 on blood monocytes, as detected by the conformation-specific single-chain antibody MAN-1. Via the same association with platelets, THP-1 monocyte adhesion to the endothelial intercellular adhesion molecule 1 (ICAM-1) is induced by ADP. P_2_Y_12_ receptor blockers prevent these ADP effects on monocytes. Interestingly, in contrast to THP-1 monocytes, THP-1 monocytes, after differentiation to macrophages, directly expressed the P_2_Y_12_ receptor and consequently ADP was found to be a potent chemoattractant. Again, P_2_Y_12_ receptor blockers antagonised this effect. Accordingly, stimulation of THP-1 macrophages with ADP caused a substantial change in gene expression pattern and upregulation of several genes associated with inflammation and atherogenesis. These data establish novel anti-inflammatory effects of P_2_Y_12_ receptor blockers on monocytes and macrophages, which are expected to contribute to cardiovascular risk reduction.

## Introduction

Vascular inflammation is the underlying cause of several acute and chronic cardiovascular diseases and is mainly promoted by platelets and leukocytes^1,2^. Activated platelets interact via their surface receptor P-selectin (CD62P) or CD40L with the leukocyte ligand P-selectin glycoprotein ligand-1 (PSGL-1) or CD40, respectively, thereby forming highly inflammatory monocyte-platelet aggregates (MPA)^3^. MPA formation induces increased expression of leukocyte adhesion molecules enabling leukocyte attachment and transmigration into the subendothelial space, where monocytes differentiate into macrophages^4,5^. Macrophages themselves release several chemoattractants, promoting the entry of new monocytes into pre-existing inflammatory atherosclerotic lesions and thus initiating a vicious cycle of vascular inflammation and monocyte recruitment, thereby maintaining the inflammatory process that drives the progression of atherosclerosis^6,7^.

Activated monocytes are the drivers of inflammation in a wide range of diseases including heart failure, acute coronary syndromes (ACS) and atherosclerosis^8–11^. Therefore, determining the monocyte activation status is of particular interest. The single-chain variable Fragment (scFv) MAN-1 binds specifically to the active conformation of the β_2_-integrin Mac-1 (CD11b/CD18; α_M_β_2_). It therefore provides a unique opportunity to monitor the activation state of circulating monocytes^12^.

The P_2_Y_12_ receptor is mainly expressed on platelets but other cell types, for example smooth muscle cells and dendritic cells have also been shown to express this receptor^13,14^. In platelets, the human P_2_Y_12_ receptor mediates activation by ADP^15,16^. ADP stimulation of the Gi-coupled P_2_Y_12_ receptor leads to activation of PI 3-kinase, Akt, ERK, Rap1b, Src family kinases and G protein-gated inwardly rectifying potassium channels resulting in activation of GPIIb/IIIa and platelet aggregation^17^.

Due to their anti-platelet effect, P_2_Y_12_ receptor blockers (e.g. clopidogrel, prasugrel, ticagrelor and cangrelor) are recommended for use in patients after percutaneous coronary intervention and in ACS to avoid thrombotic complications, particularly stent thrombosis^18^. Recent data indicate that P_2_Y_12_ receptor blockers, in addition to anti-thrombotic effects, also have anti-inflammatory effects^19–22^. The mechanisms, however, by which P_2_Y_12_ receptor blockers mediate these anti-inflammatory effects are currently poorly understood^18,23^.

This study aimed to investigate the anti-inflammatory effects of P_2_Y_12_ receptor blockers on monocytes and macrophages.

## Results

### ADP activates monocytes in a platelet-dependent manner

**Figure 1:**
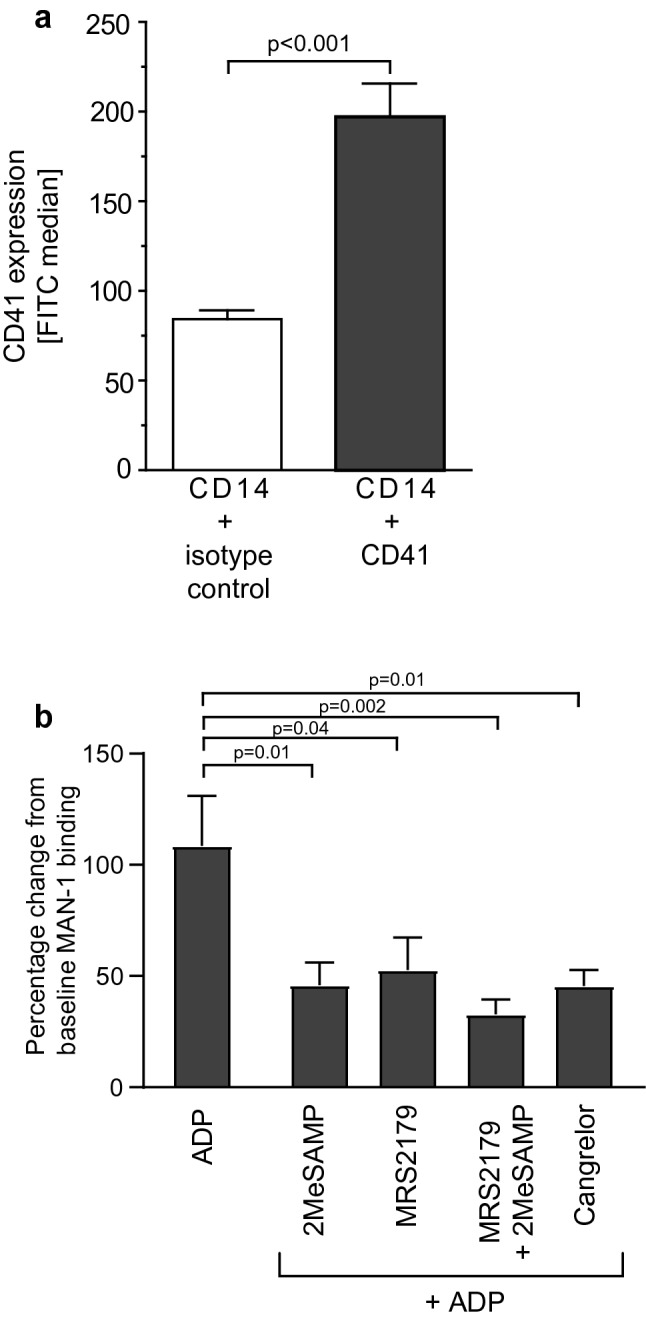
Monocytes are activated by ADP and inhibited by P_2_Y_1_ and P_2_Y_12_ receptor blockers, mediated by platelets in monocyte platelet aggregates. (**a**) CD14^+^/CD41^+^ MPA were detected by flow cytometry in whole blood from healthy volunteers. The FITC median of all CD14^+^ monocytes stained with an anti-CD41 FITC antibody is presented. *p*-values were calculated by a Student’s t-test, n = 8. (**b**) Using flow cytometry, Mac-1 activation on monocytes was determined using the activation-specific anti-Mac-1 scFv MAN-1. MAN-1 binding is presented as percentage change from baseline which was calculated as shown in the “Methods” section. The median FITC of all CD14^+^ monocytes was recorded. *p*-values were calculated by one-way ANOVA followed by Tukey’s multiple comparison post-test, n = 6. Data are presented as mean ± SEM.

We hypothesised that monocyte activation is mediated by ADP indirectly via platelets attached to monocytes. Using flow cytometry, platelet–monocyte complexes could be detected in the blood of healthy volunteers (median CD14-Iso FITC vs. median CD14-CD41: 84 ± 5 vs. 197 ± 19, *p* < 0.001, Fig. 1a). The percentage of MPA of CD14^+^ monocytes in lysed whole blood from healthy volunteers was approximately 7.8% (Supplementary Fig. S1). Additionally, assessment of CD62P expression using flow cytometry showed that platelets were not artificially activated as individual platelets but are activated as components of monocyte platelet aggregates (MPA) (Supplementary Fig. S2). We therefore assumed that a proportion of peripheral blood monocytes form MPA even in the unstimulated blood of healthy volunteers. To investigate whether blood monocytes (assumed to be within MPA) can be activated via a platelet-dependent pathway, whole blood was stimulated with ADP (F_c_ 20 μM) and Mac-1 activation on CD14^+^ monocytes was assessed in flow cytometry using the activation-specific single-chain antibody MAN-1 (Fig. 1b). Addition of ADP to whole blood increased levels of activated Mac-1 on monocytes (percentage change from baseline MAN-1 binding: ADP-activated monocytes vs unstimulated monocytes: 108.6 ± 22.6 vs. 0.0 ± 0.0, *p* < 0.001). This effect was inhibited after pre-incubation of whole blood with different P_2_Y_12_ receptor blockers (percentage change from baseline MAN-1 binding: ADP-activated monocytes vs. ADP-activated monocytes + 2MeSAMP (F_c_ 100 μM): 108.6 ± 22.6 vs. 46.05 ± 10.1, *p* = 0.01; ADP-activated monocytes vs. ADP-activated monocytes + cangrelor (F_c_ 100 nm): 108.6 ± 22.6 vs. 45.6 ± 7.10, *p* = 0.01; Fig. 1b). To assess whether this effect is specific for P_2_Y_12_ receptors, the effect of the P_2_Y_1_ receptor blocker MRS2179 (F_c_ 100 µM) on MPA formation was assessed, and it was found that this also inhibited monocyte activation (percentage change from baseline MAN-1 binding: ADP-activated monocytes vs. ADP-activated monocytes + MRS2179: 108.6 ± 22.6 vs. 52.66 ± 14.7, *p* = 0.04; Fig. 1b).

### Platelet–monocyte aggregates circulate in blood of healthy volunteers and are increased in patients with CAD and ACS

**Figure 2:**
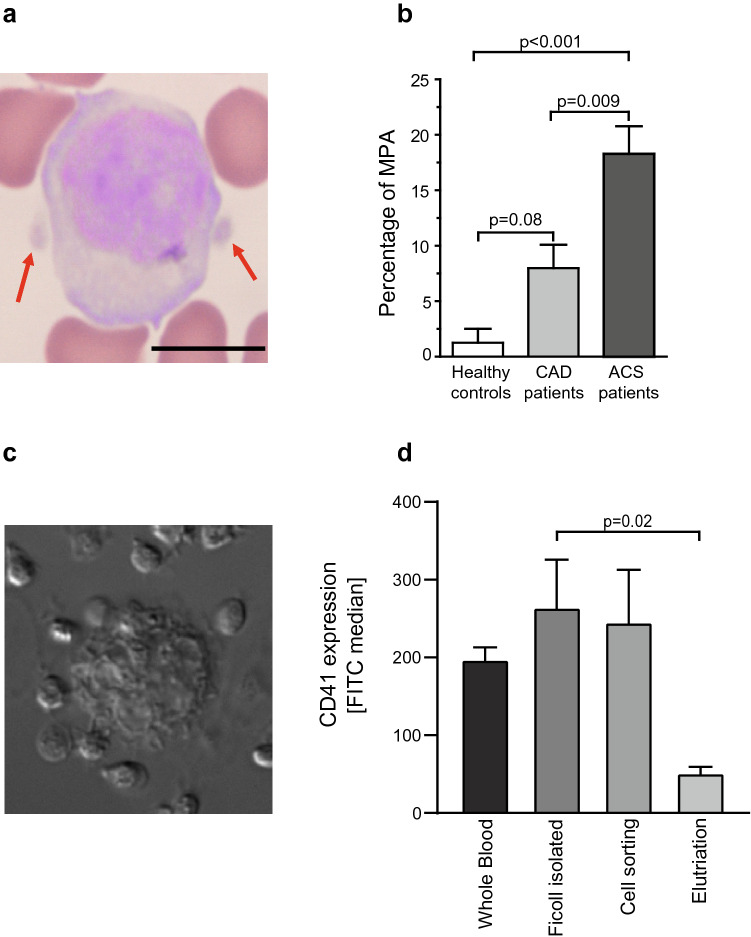
Presence of monocyte platelet aggregates in circulating blood of healthy volunteers and patients with CAD and ACS using different monocyte isolation methods. To determine whether MPA are present in all monocytes isolated from whole blood, levels of MPA were assessed using different methods as described in the “Methods” section. (**a**) Platelets adherent to monocytes were found in small numbers in the blood smears of healthy subjects. The light microscopic image depicts a monocyte with two adhering platelets indicated by arrows. Scale bar = 10 µm. (**b**) Quantification of MPA in percentage of all monocytes in blood smears demonstrated increasing percentages of monocytes with adherent platelets in healthy volunteers, CAD and patients with acute coronary syndrome. n = 5 per group. (**c**) Microscopic image of a representative monocyte isolated by Ficoll density gradient centrifugation at 1000× magnification using an inverted microscope. (**d**) MPA were found in healthy subjects after isolation of monocytes using different isolation methods. n ≥ 3, *p*-values were calculated using one-way ANOVA.

Several techniques were used to test for the presence of MPA in circulating blood (see “Methods” section): 1. blood smear analysis (Fig. 2a,b); 2. whole-blood flow cytometry, 3. Ficoll density gradient; 4. a cell sorter; 5. elutriation. Levels of MPA using techniques/methods 2–5 were analysed by flow cytometry. Notably, platelets were present on monocytes isolated from whole blood using all methods.

The percentage of MPA in blood smears was higher in patients with stable coronary artery disease (CAD) and higher yet in patients with ACS compared to smears from healthy volunteers (healthy controls vs. CAD vs. ACS: 1.3 ± 1.5 vs. 8.0 ± 2.1 vs. 18.3 ± 2.4, *p* < 0.001) (Fig. 2b), which is in line with the literature^24,25^. The levels of MPA depends on the isolation method (FITC median: whole blood vs. Ficoll-isolated (Fig. 2c,d) vs. cell-sorted vs. elutriation: 197.0 ± 18.7 vs. 264.0 ± 63.6 vs. 245.0 ± 70.4 vs. 51.0 ± 11.0; *p* = 0.02 for Ficoll-isolated vs. elutriation) (Fig. 2a–d).

### MPA formation and P_2_Y_12_ stimulation facilitate monocyte adhesion to ICAM-1 under flow conditions

**Figure 3:**
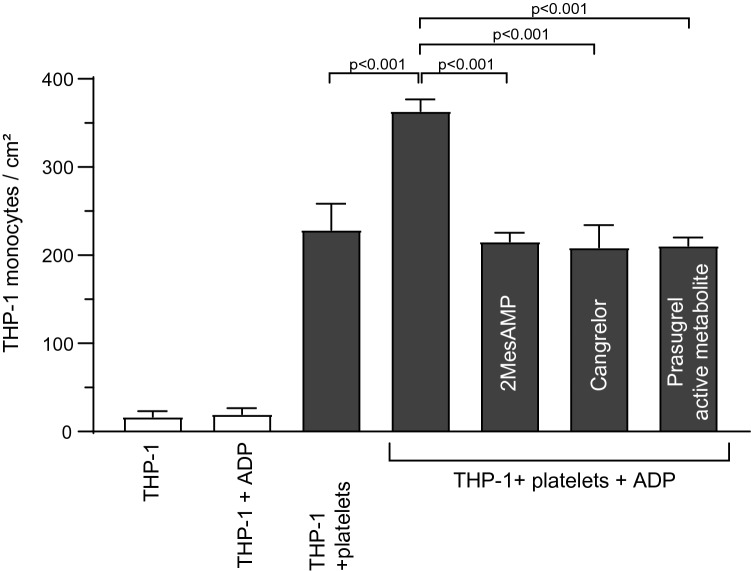
ADP-stimulated monocyte platelet aggregates bind to ICAM-1 under shear flow. Flow chamber experiments were conducted as described in the “Methods” section. The number of adhering THP-1 monocytes/cm^2^ as determined under a microscope using a Neubauer chamber is presented. THP-1 cells alone either with or without ADP stimulation did not bind to ICAM-1. However, THP-1 monocytes pre-incubated with platelets demonstrated binding to ICAM-1 and this binding was increased with ADP stimulation. This increase could be inhibited by the addition of P_2_Y_12_ receptor blockers (2MesAMP, cangrelor and prasugrel active metabolite). n = 5, data are presented as mean ± SEM. *p*-values were calculated using one-way ANOVA and a Tukey’s multiple comparison post-test.

Adhesion of monocytes to endothelial cells and subendothelial matrices is a pivotal initial step in vascular inflammation. Using flow-chamber experiments, we assessed whether monocyte adhesion to ICAM-1 is mediated by P_2_Y_12_ receptors on platelets bound to monocytes and whether it can be inhibited by P_2_Y_12_ receptor blockers (Fig. 3). Under physiological shear-stress conditions, non-activated monocytic cells did not adhere to ICAM-1 in relevant numbers, either with or without ADP stimulation (THP-1 monocytes/cm^2^: THP-1 monocytes vs. THP-1 monocytes + ADP: 16.9 + 6.3 vs. 19.9 ± 6.5, *p* > 0.99). However, adhesion to ICAM-1 was significantly increased for THP-1 monocytes pre-incubated with washed platelets, particularly when they were stimulated with ADP (THP-1 monocytes/cm^2^: THP-1 monocytes + platelets vs. THP-1 monocytes + platelets + ADP: 228.9 ± 29.5 vs. 363.4 ± 13.3, *p* < 0.001). Pre-treatment of ADP-stimulated THP-1 monocytes + platelets with prasugrel (active metabolite), cangrelor or 2MeSAMP reduced adhesion of aggregates to ICAM-1 (THP-1 monocytes + platelets + ADP vs. THP-1 monocytes + platelets + ADP + prasugrel active metabolite vs. THP-1 monocytes + platelets + ADP + cangrelor vs. THP-1 monocytes + platelets + ADP + 2MeSAMP: 363.4 ± 13.3 vs. 210.8 ± 9.5 vs. 208.7 ± 25.6 vs. 215.4 ± 10.1, *p* < 0.001).

### Macrophages, but not peripheral blood monocytes, express P_2_Y_12_ receptors

**Figure 4:**
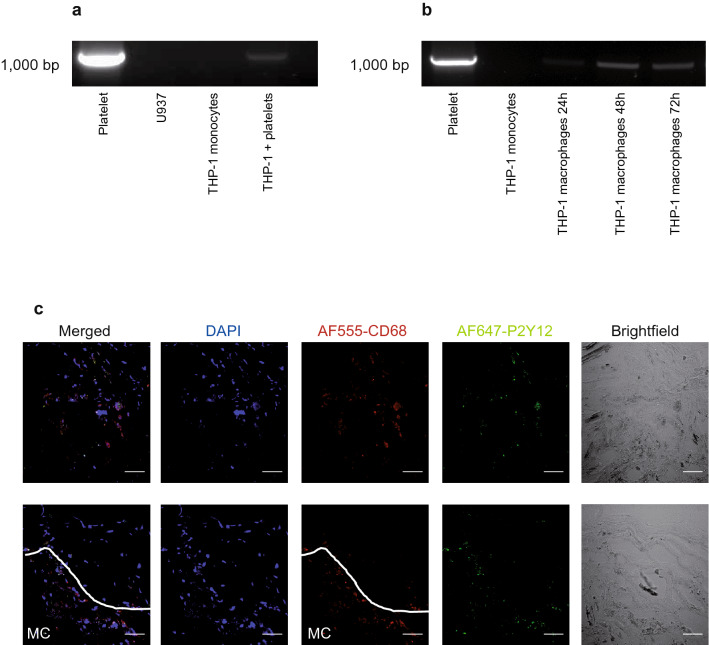
P_2_Y_12_ receptor mRNA is expressed in differentiated THP-1 macrophages but not naive THP-1 monocytic cells and P_2_Y_12_ receptor antigen is present on CD68 positive macrophages in human atherosclerotic plaque. 1% agarose gel showing RT-PCR results using primers specific for the P_2_Y_12_ receptor as described in the “Methods” section. Depicted gels have been cropped and the DNA standard is shown to the left of the gel (1000 bp). Uncropped gels can be found in Supplementary Figures S3, 4. (**a**) Monocytic cell lines (THP-1 and U937) did not express P_2_Y_12_ receptor. P_2_Y_12_ receptor mRNA expression could be detected after pre-incubation of THP-1 monocytes with washed platelets (band “THP- 1 + platelets”). (**b**) Once differentiated to macrophages, THP-1-derived macrophages expressed P_2_Y_12_ receptor mRNA at increasing levels 24, 48 and 72 h after induction of macrophage differentiation. (**c**) Anti-CD68 and anti-P_2_Y_12_ immunofluorescence on human carotid atherosclerotic plaques obtained by carotid endarterectomy. Preparation and staining were performed as described in the “Methods” section. The different channels are named at the top of the images (“Merged”, “DAPI” etc.). Immunofluorescence revealed that CD68^+^ macrophages within the atherosclerotic plaque also expressed the P_2_Y_12_ receptor. Scale bar = 50 µm. MC = CD68^+^ macrophage concentrated area.

P_2_Y_12_ receptor expression of monocytes was assessed with RT-PCR (see “Methods” section). To assess P_2_Y_12_ receptor expression in peripheral blood monocytes without the risk of platelet contamination, the monocytic cell lines THP-1 and U937 were used as cell models for monocytes. As shown in Fig. 4a, P2Y_12_ receptor mRNA could not be detected in both monocytic cell lines. As expected, in THP-1 monocytes pre-incubated with washed platelets, P_2_Y_12_ receptor mRNA could be detected. However, after differentiation of THP-1 monocytes into macrophages following stimulation with phorbol 12-myristate 13-acetate (PMA), P_2_Y_12_ receptor mRNA could be detected (Fig. 4b).

Furthermore, using immunofluorescence microscopy, P_2_Y_12_ receptor expression was confirmed on CD68^+^ macrophages in human atherosclerotic plaques obtained by carotid endarterectomy (CEA), indicating that P_2_Y_12_ receptor expression on macrophages also occurs in vivo (Fig. 4c).

### ADP induces IL-1, IL-8 and MMP-9 expression in macrophages

**Figure 5:**
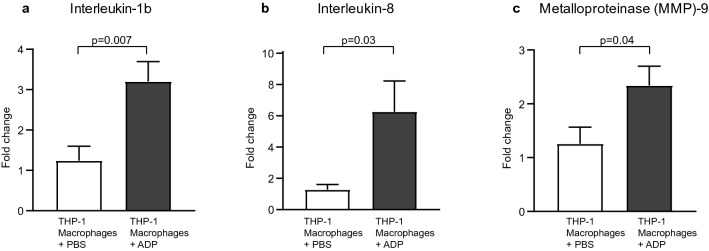
ADP stimulation induces expression of pro-inflammatory genes in P_2_Y_12_ receptor expressing THP-1 macrophages. Stimulation of THP-1 macrophages with ADP or PBS, RNA isolation and qRT-PCR with primers for IL-1b, IL-8 and MMP-9 was performed as described in the “Methods” section. ADP stimulation resulted in upregulation of the pro-inflammatory genes such as IL-1b (**a**), IL-8 (**b**) and MMP-9 (**c**). Fold change of expression in relation to the housekeeping gene is presented as 2^-∆Ct^ and *p*-values were calculated by an unpaired Student’s t-test, n = 3. Data are presented as mean ± SEM.

Having shown that THP-1 macrophages express P_2_Y_12_, we hypothesised that ADP acts as a pro-inflammatory stimulus on macrophages. 24 h of ADP stimulation led to significant upregulation of IL-1b (Fig. 5a), IL-8 (Fig. 5b), and MMP-9 (Fig. 5c) expression in macrophages, as determined by quantitative RT-PCR (fold change THP-1 macrophages + phosphate-buffered saline (PBS) vs. THP-1 macrophages + ADP: IL-1b 1.23 ± 0.36 vs. 3.2 ± 0.49, *p* = 0.007; IL-8 1.27 ± 0.33 vs. 6.26 ± 1.97 *p* = 0.03; MMP-9 1.26 ± 0.31 vs. 2.34 ± 0.36, *p* = 0.04).

### Pro-inflammatory gene expression in ADP-stimulated macrophages

**Figure 6:**
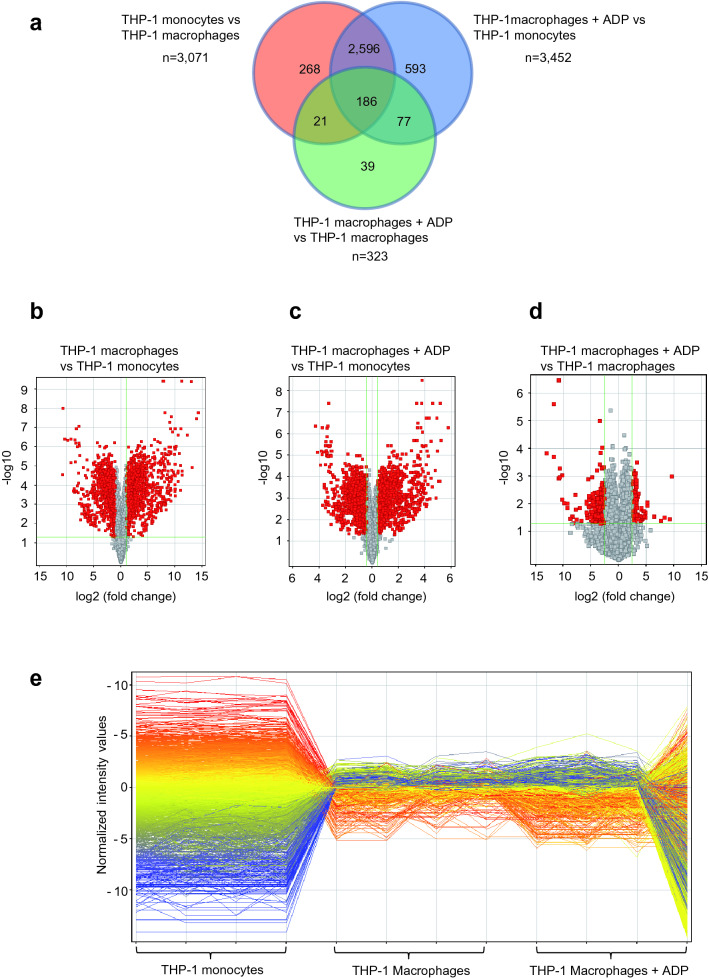
ADP stimulation of P_2_Y_12_-expressing THP-1 macrophages upregulates inflammatory genes. RNA was isolated from THP-1 monocytes, THP-1 monocytes which had been differentiated to macrophages (= THP-1 macrophages) and THP-1 macrophages which had been stimulated with ADP. RNA microarrays were performed as described in the “Methods” section. Gene expression of THP-1 macrophages stimulated with ADP was compared to those of THP-1 monocytes and unstimulated THP-1 macrophages. A Venn diagram (**a**) and several volcano blots (**b**–**d**) were created to illustrate differential gene expression between groups. (**a**) On the Venn diagram, n = x indicates the number of differentially expressed genes between groups and the numbers in the circles indicate genes that are shared by different groups. (**b**) 3071 genes in total were differentially expressed between THP-1 monocytes and unstimulated THP-1 macrophages, many of which were upregulated in THP-1 macrophages. (**c**) 3452 genes were differentially expressed between ADP-stimulated THP-1 macrophages and THP-1 monocytes; and (**d**) 323 genes were differentially expressed between ADP-stimulated THP-1 macrophages and unstimulated THP-1 macrophages. (**e**) The baseline transformation plot shows normalised intensity values of differentially expressed genes in the different groups: THP-1 monocytes, THP-1 macrophages and THP-1 macrophages + ADP. Differentiation of THP-1 monocytes to macrophages led to a significant reduction in the diversity of expressed genes. The diversity of genes in THP-1 macrophages compared to ADP-stimulated THP-1 macrophages was only slightly increased.

To investigate whether the P_2_Y_12_ receptor expressed on THP-1 macrophages is involved in macrophage gene regulation, THP-1 macrophages were stimulated with ADP and gene expression was compared to PBS-treated THP-1 macrophages and THP-1 monocytes. As shown in a Venn diagram (see Fig. 6), mRNA profiling revealed that 3071 genes were differentially expressed between THP-1 monocytes and THP-1 macrophages, 3452 genes were differentially expressed between ADP-stimulated THP-1 macrophages and THP-1 monocytes, and 323 genes were differentially expressed between ADP-stimulated THP-1 macrophages and unstimulated THP-1 macrophages (Fig. 6a). THP-1 macrophages in comparison to THP-1 monocytes showed several genes that were highly upregulated (Fig. 6b). The same trend was found in ADP-stimulated macrophages vs. THP-1 monocytes (Fig. 6c). A lower number of differentially regulated genes was found when comparing ADP-stimulated THP-1 macrophages to THP-1 macrophages (Fig. 6d). As shown in Fig. 6e, THP-1 monocytes expressed a more diverse pattern of genes than THP-1 macrophages. However, stimulation of macrophages with ADP resulted in a slight increase in differentially regulated genes in comparison to macrophages without ADP treatment. These comprised 323 genes, many of them involved in pro-inflammation, such as MCPIP1, TRAF1 and ECGF1. These data indicate significant functional relevance of the macrophage P_2_Y_12_ receptor in inflammatory macrophage gene regulation. Examples of upregulated genes in ADP-stimulated THP-1 macrophages vs. PBS treated THP-1 macrophages relevant to vascular inflammation and pathogenesis of atherosclerosis are presented in Supplementary Table S1. The full list of differentially regulated genes is presented as Supplementary Table S2.

### ADP is a chemoattractant for P_2_Y_12_-expressing macrophages

**Figure 7:**
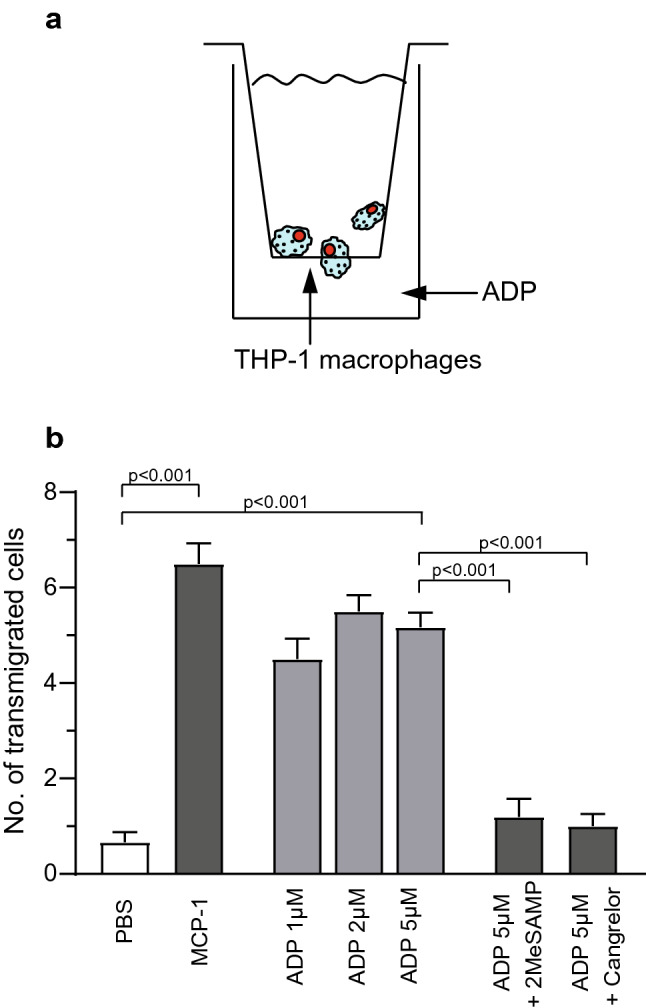
ADP is a chemoattractant for P_2_Y_12_ expressing THP-1 macrophages. (**a**) Principle of the transwell assay. More information can be found in the “Methods” section. (**b**) It was found that THP-1 macrophages showed increased migration towards ADP compared to PBS. This migratory behaviour was inhibited by different P_2_Y_12_ receptor blockers. MCP-1 served as a positive control. n = 5, data are presented as mean ± SEM. *p*-values were calculated by one-way ANOVA.

Migration into inflamed tissue areas is a key function of macrophages. Hence, it was investigated whether ADP acts as a chemoattractant for THP-1-macrophages, for which P_2_Y_12_ expression was demonstrated earlier. Only very little migration was observed towards the negative control (PBS). THP-1 macrophages migrated towards ADP (Fig. 7) and migration towards ADP was inhibited by pre-incubation with the P_2_Y_12_ receptor blockers 2MeSAMP and cangrelor (number of transmigrated cells 5 µM ADP vs. PBS: 5.2 ± 0.3 vs. 0.7 ± 0.2, *p* < 0.001; 5 µM ADP vs. 5 µM ADP + 2MeSAMP vs. 5 µM ADP + cangrelor: 5.2 ± 0.3 vs. 1.2 ± 0.4 vs. 1.0 ± 0.3, *p* < 0.001). Strong migration of THP-1 macrophages was also observed towards the positive control ‘monocyte chemoattractant protein 1’ (MCP1; number of transmigrated cells MCP-1 vs. PBS: 6.5 ± 0.4 vs. 0.7 ± 0.2, *p* < 0.001).

## Discussion

The present study shows that P_2_Y_12_ receptor blockers inhibit monocyte and macrophage function, and thus have direct effects on inflammation and immunity. On circulating monocytes, P_2_Y_12_ receptor blockers act indirectly, in a platelet-dependent manner. Furthermore, they inhibit binding of THP-1 monocytes incubated with platelets to ICAM-1 under shear stress, a step that is vital in the development and progression of vascular wall inflammation. On differentiated macrophages, P_2_Y_12_ receptor blockers act directly by binding to the P_2_Y_12_ receptors expressed upon differentiation. Furthermore, stimulation of macrophages with ADP significantly increases expression of pro-inflammatory genes, many of which are involved in the development and progression of atherosclerosis. Moreover, ADP is identified as a potent chemoattractant for P_2_Y_12_ receptor–expressing macrophages, a mechanism potentially contributing to macrophage accumulation at the sites of ADP-rich necrotic areas and ultimately unstable atherosclerotic plaques.

ADP is one of the physiologically most important platelet agonists and it is evident that the P_2_Y_12_ receptor is strongly involved in platelet activation and aggregation. Due to their anti-platelet effects, P_2_Y_12_ receptor blockers are among the most commonly prescribed drugs for patients with cardiovascular diseases. However, it has been suggested that P_2_Y_12_ receptor blockers not only exert an anti-thrombotic effect, but also attenuate vascular inflammation^18,20^.

To investigate whether P_2_Y_12_ receptor blockers have any impact on monocyte activation, and thereby vascular inflammation, Mac-1 activation on monocytes in whole blood was assessed in response to ADP stimulation and in the presence of P_2_Y_12_ receptor blockers. Mac-1 is a leukocyte β_2_ integrin that in its active receptor conformation is responsible for multiple ligand–receptor interactions such as binding to the endothelial ligand ICAM-1, GPIb, CD40L, vitronectin, fibrinogen, heparin and the protein C receptor and therefore is involved in multiple acute and chronic cardiovascular diseases^26–32^.

To the best of our knowledge, the present study is the first to demonstrate that addition of ADP to whole blood induces Mac-1 activation on circulating monocytes, which can be inhibited by P_2_Y_12_ receptor blockers. ADP stimulation of whole blood also significantly increases numbers of MPA^33,34^. We provide data indicating that the anti-inflammatory effect of P_2_Y_12_ receptor blockers on circulating blood monocytes is platelet-dependent via MPA formation. Increased CD62P on ADP-activated platelets binding to PSGL-1 on monocytes may be one of the underlying mechanisms behind monocyte activation via ADP, as other groups have described monocyte activation through platelet CD62P leading to increased expression of pro-inflammatory cytokines, e.g. TNFα, IL-1β and IL-6^35^.

In this line of evidence, previous reports suggested that MPA were increased in patients with myocardial infarction (MI) versus stable coronary artery disease^36,37^. Reduced levels of MPA were described in patients treated with the P_2_Y_12_ receptor blockers prasugrel or clopidogrel. Formation of MPA is facilitated by PSGL-1 on monocytes binding to CD62P on platelets. ADP stimulation induces increased expression of CD62P on the platelet surface. Inhibition of ADP-triggered CD62P expression by P_2_Y_12_ receptor blockers may therefore also be one of the mechanisms behind reduced MPA levels in patients receiving prasugrel or clopidogrel^23,38^.

The impact of CD62P on vascular inflammation has been confirmed by a recent study investigating patients with COVID-19^39^. Increased MPA formation correlated with poor outcomes in these patients. Moreover, it was found that platelets triggered tissue factor expression in monocytes through increased CD62P expression, thereby contributing to microthrombotic and thromboembolic complications^39,40^.

P_2_Y_12_ receptor blockers may also lead to fewer MPA adhering to the arterial vessel wall in vivo either by reducing MPA formation or, more likely, by indirectly decreasing Mac-1 activation on monocytes via platelets. This hypothesis is supported by our flow-chamber data, in which the adhesion of MPA to the Mac-1 ligand ICAM-1 was found to be increased by ADP, whereas pre-treatment of MPA with P_2_Y_12_ receptor blockers such as prasugrel, cangrelor and 2MeSAMP reversed this effect. However, we cannot exclude that other leukocyte integrins, such as LFA-1, may also be activated by ADP and be involved in adhesion of MPA to the vessel wall.

We aimed to assess the expression of P_2_Y_12_ receptors in monocytes and macrophages. RT-PCR has been frequently used to determine the expression levels of purinergic receptors in blood leukocytes^41,42^. Using the five common methods of assessing monocytes with flow cytometry or microscopy (elutriation, cell sorting, blood smear, Ficoll density gradient, whole-blood flow cytometry), we demonstrated that a significant proportion of monocytes carry platelets on their surface, even if isolated with the least activating purification methods, elutriation and cell sorting. This is in line with previous reports where platelet–monocyte complexes were present even in healthy cohorts at relevant levels^43^. Therefore, analysis of monocyte mRNA expression is at high risk of contamination with platelet mRNA such as the P_2_Y_12_ receptor mRNA. To assess gene expression in monocytes without risk of platelet contamination, the expression of P_2_Y_12_ was investigated in two monocytic cell lines (THP-1, U937). Neither THP-1 cells nor U937 cells expressed the P_2_Y_12_ receptor. Hence, the activation of blood monocytes is most likely caused by an indirect mechanism via platelets attached to the monocyte surface. It is reasonable to assume that this P_2_Y_12_ receptor–mediated anti-inflammatory effect contributes to the improved outcomes observed in ACS patients being treated with P_2_Y_12_ receptor blockers^44^.

Interestingly, Micklewright et al. recently reported expression of P_2_Y_12_ receptors in whole blood monocytes and THP-1 cells. The finding of P_2_Y_12_ receptor presence in CD14^+^ monocyte preparations may, however, be the result of platelet monocyte aggregate formation and thus is in accordance to our findings. The fact that the authors found P_2_Y_12_ receptor expression on THP-1 monocytes, in contrast to our findings, may reflect the variability in expression often to be found in immortalized cell lines in culture. A stable cell line such as THP-1, may well have developed a selection towards P_2_Y_12_ expression depending on the culture conditions. In contrast, our experimental set-up is based on a more reliable comparison of our THP-1 cells before and after differentiation by PMA. Our data strongly indicates that P_2_Y_12_ expression is upregulated with the differentiation of monocytes to macrophages as the main finding. In fact, in regards to circulating monocytes, previous studies are also supportive of our data. For example, it was reported that native monocytes do not express the P_2_Y_12_ receptor in contrast to differentiated macrophages^45^. Moreover, an earlier study has shown that blood monocytes did not express the P_2_Y_12_ receptor, whereas microglia cells, which are similar to resident macrophages, expressed the P_2_Y_12_ receptor^46^.

Once attached to endothelium covering atherosclerotic lesions, monocytes are recruited into the subendothelial space and can differentiate into highly inflammatory macrophages involved in the development of the necrotic core of unstable atherosclerotic plaques. Rupture of unstable atherosclerotic plaques can lead to fatal consequences such as MI and stroke^47^. Hence, anti-inflammatory drugs that inhibit plaque inflammation and provide plaque stabilisation are of particular interest.

As we demonstrated P_2_Y_12_ receptor expression in macrophages and that these cells are located within human atherosclerotic plaques, we suggest these macrophages may be targeted directly by P_2_Y_12_ receptor blockers, leading to plaque stabilisation in vivo. Our hypothesis is supported by data in the literature. For example, different groups have reported a reduction in plaque inflammation and smaller plaque sizes in animals treated with P_2_Y_12_ receptor blockers^48–51^.

Micklewright et al. showed a significant increase of intracellular calcium levels after stimulation of THP-1 cells with ADP, indicating cellular activation and levels were attenuated after P_2_Y_12_ receptor blockade with ticagrelor^52^. In contrast, our study demonstrated an indirect mechanism of monocyte activation in whole blood by ADP: via platelets. Moreover, we used the unique activation-specific single chain variable fragment “MAN-1” to specifically detect monocyte activation induced by ADP.

Pro-atherogenic and pro-inflammatory gene expression in response to ADP stimulation were however not investigated by Micklewright et al. This was addressed in our study for P_2_Y_12_ receptor expressing THP-1 derived macrophages using qRT-PCR followed by mRNA profiling. The rupture of vulnerable atherosclerotic plaques is the most frequent cause of MI. Matrix metalloproteinases produced by macrophages such as MMP-9 play a pivotal role in the aetiology of plaque rupture, as these enzymes break down extracellular matrices such as fibrillar collagen, thereby reducing the stability of atherosclerotic plaques^53–55^. The importance of MMP-9 is highlighted in the finding that its plasma level is a predictor of cardiovascular death^56^. This observation is in accordance with our data, as we found that ADP stimulation of P_2_Y_12_-expressing macrophages significantly increased MMP-9 mRNA levels. This indicates that by inhibition of MMP-9 production, P_2_Y_12_ receptor blockers may contribute to plaque stabilisation.

Using mRNA microarray technology, we found that ADP significantly upregulates over 300 genes in THP-1 macrophages, several of which are known to be involved in endothelial inflammation and progression of atherosclerosis. For example, TGFβ_3_ and TNFα were upregulated in macrophages treated with ADP and have been shown to promote negative remodelling and plaque instability^57^. Moreover, TNF receptor associated factor (TRAF)-1, VEGFA and syndecan-4 were also increased in macrophages treated with ADP and have been shown to play a major role in the formation and development and progression of the atherosclerotic plaque^58–60^.

P_2_Y_12_ receptor blockade has been shown to reduce ADP-induced upregulation of inflammatory genes. For example, in a mouse model of myocardial fibrosis and inflammation by angiotensin II infusion, clopidogrel treatment significantly reduced the levels of IL-1β and TFGβ^61^. Moreover, in an lipopolysaccharide-induced systemic inflammation rat model, pre-treatment with clopidogrel significantly reduced levels of TNFα and IL-6^62^. Heim et al*.* demonstrated significantly reduced levels of MCP-1 and PDGFβ in ApoE^-/-^ mice on a high-fat diet receiving clopidogrel compared to controls^49^. These data indicate that P_2_Y_12_ receptor blockade not only prevents thrombotic complications such as stent thrombosis, but also reduces inflammation and progression of atherosclerosis, most likely by affecting monocyte/macrophage function. However, clopidogrel, ticagrelor and prasugrel may also be exerting their anti-inflammatory effects by targeting other P_2_Y_12_ receptor–expressing cells in the atherosclerotic plaque such as smooth muscle cells^63,64^.

In addition to demonstrating P_2_Y_12_ receptor expression in macrophages in the atherosclerotic plaque of carotid endarterectomies, we have demonstrated that ADP acts as a strong chemoattractant for macrophages and that migration towards higher ADP concentrations is a P_2_Y_12_-dependent effect. A similar observation was made by Micklewright et al. who showed that ticagrelor inhibits THP-1 monocyte migration towards ADP^52^. This mechanism could also potentially occur in the human atherosclerotic plaque. Our data is supported by a study of Dunzendorfer et al*.*, who found that subjects treated with clopidogrel showed less ex vivo adhesion of monocytes on endothelial cells and reduced chemokinesis of monocytes compared to subjects not treated with clopidogrel^65^. Recently, migration of macrophages towards ADP secreted by dying melanoma cells was shown by Kloss et al*.*^45^, expanding the role of the P_2_Y_12_ receptor in macrophages to cancer pathophysiology. In this line of evidence, a group recently published a nested case–control study indicating that clopidogrel treatment reduced the occurrence of colorectal cancer^66^.

It remains to be elucidated which P_2_Y_12_ receptor to use when targeting inflammatory diseases. Prasugrel and ticagrelor inhibit platelet function more efficiently than clopidogrel. However, prasugrel and ticagrelor also carry an increased risk of bleeding events^23^.

This study is not without limitations. Although we found that pre-incubation of whole blood with the P_2_Y_1_ receptor blocker MRS2179 before addition of ADP reduced monocyte activation, we focused our study on P_2_Y_12_ receptor blockers based on their central clinical relevance and anti-inflammatory effects. It is possible, however, that the P_2_Y_1_ receptor also plays a role in monocyte activation after addition of ADP to whole blood. As most assays were conducted with THP-1 monocytes and THP-1 monocyte-derived macrophages, we cannot exclude that the artificial cell culture environment may have favoured, for example, P_2_Y_12_ receptor expression in THP-1 macrophages. This strategy was chosen since significant “platelet contamination” was expected if native monocytes derived from whole blood had been used. Moreover, no specific P_2_Y_12_ receptor blocker was used for the gene expression studies. Therefore, we cannot exclude that other P_2_Y receptors may contribute to ADP-induced gene up- or downregulation. Additionally, as a specific staining for platelet antigens in the atherosclerotic plaques was not performed, it is possible that some of the P_2_Y_12_ receptor expression detected in the atherosclerotic plaque is due to the presence of platelets on macrophages.

## Conclusion

Circulating monocytes in the peripheral blood seem not to express the P_2_Y_12_ receptor. However, P_2_Y_12_ receptor blockers act indirectly in a platelet-dependent manner on monocytes as anti-inflammatory agents. In contrast, differentiated macrophages express the P_2_Y_12_ receptor, their protein expression is altered by ADP stimulation and they migrate towards ADP. Macrophage migratory functions can be directly inhibited by P_2_Y_12_ receptor blockers, reflecting direct anti-inflammatory and atheroprotective effects.

## Methods

### Patient samples

ACS patients were defined according to the NSTEMI guidelines of the European Society of Cardiology. Patients were eligible if they presented with new onset of thoracic pain and had elevated levels of high-sensitivity troponin T. CAD patients presented with stable angina and did not have increased levels of troponin T. After diagnosis and before coronary angiography, citrated whole blood was taken by antecubital vein puncture, drawn slowly to prevent artificial platelet activation, and immediately transported to the laboratory for blood smear analysis. Blood from healthy volunteers was also taken and analysed as described below. Healthy volunteers had no underlying conditions and had not taken any medication in the last 14 days. Measurements of patient samples were performed in accordance with the Helsinki Declaration (2008). Informed consent was obtained from all patients and healthy volunteers prior to inclusion in this study. The study was approved by the Alfred Hospital Ethics Committee.

Human carotid endarterectomy (CEA) specimens were collected immediately after being removed in the operating theatre from patients who presented to the Alfred Hospital, Melbourne, Australia with clinical indications for CEA. This project was approved by the Alfred Hospital Ethics Committee and all participants signed the informed consent form.

### Quantification of platelet–monocyte aggregates

Due to methodological issues (e.g., blood taking, centrifugation) monocytes are often pre-activated ex vivo*,* leading to artificial MPA formation and reduced specificity and sensitivity of MPA as inflammatory biomarkers^1^. We assessed the impact of the 5 common monocyte isolation techniques on artificial ex vivo MPA formation. In brief, MPA were:

1. Isolated by Ficoll gradient centrifugation (Ficoll-Paque™, GE Healthcare, USA) and assessed in fluorescence-activated cell sorting (FACS). 15 ml Ficoll was covered with 20 ml citrated blood (which had been diluted 1:1 with PBS + Ca^2+^/Mg^2+^) and centrifuged for 20 min (RT, 160 g, no brake). After discarding the upper 10 ml of platelet-rich solution, a second centrifugation step was performed (RT, 20 min, 350 g, no brake) and the monocytes contained in the turbid phase were extracted and washed twice with 6 ml PBS. Cells were counted in a Neubauer chamber and adjusted to a concentration of 500,000/ml with PBS.
2. Sorted from lysed whole blood using a cell sorter (FACS Aria, BD, USA) with consecutive FACS analysis. Cell sorting was done after red blood cell lysis of whole venous blood and staining of the remaining cells with 10 μl anti-CD14-PE (Beckman Coulter, USA; 15 min, 4 °C). Cells were sorted with a 3-laser, 9-colour cell sorter system (FACSAria™ Cellsorter, Becton Dickinson, USA) that sorted only CD14-positive cells, that is, monocytes. Cells were adjusted to 500,000/ml using PBS. MPA on sorted CD14^+^ monocytes were then quantified by flow cytometry as described below.
3. Separated from other blood cells by monocyte elutriation. Elutriation was performed with peripheral blood mononuclear cells (PBMC) that had previously been isolated by Ficoll density gradient centrifugation as described above. PBMC were centrifuged in an elutriation buffer containing PBS with 1% FCS and 2 mM EDTA in a Sanderson chamber (JE-5.0 rotor; 2500 rpm, 12 °C). After a gradual increase in the flow rate, monocytes were obtained at a flow rate of 18–20 ml/min. Elutriated cells were checked for size and granularity by flow cytometry and adjusted to a concentration of 500,000 cells/ml using PBS. MPA were then quantified by flow cytometry as described below.
4. Counted using light microscopy in blood smears stained with Pappenheim’s staining. At least 100 monocytes were counted per experiment.
5. Measured in whole blood using flow cytometry after red blood cell lysis (see “Flow cytometry” section).

### Flow cytometry

Activation of monocytes was assessed by flow cytometry (FACS Canto II, BD, USA) using a previously generated single-chain antibody (MAN-1) that specifically binds to the activated receptor conformation of Mac-1 and thereby enables the assessment of monocyte activation^12^. For flow cytometric analysis, citrated whole-blood samples were drawn slowly with a 21 G butterfly needle (Sarstedt, Germany) from the cubital veins of healthy donors as described above. To assess monocyte activation in FACS, 100 µl whole blood was incubated with ADP (F_c_ 20 μM; Sigma Aldrich, USA) or PBS + Ca^2+^/Mg^2+^ (negative control) for 15 min at 37 °C.

In samples in which the effect of P_2_Y_12_ or P_2_Y_1_ receptor blockers was assessed, whole blood was pre-incubated with 2MeSAMP (P_2_Y_12_ receptor blocker, F_c_ 100 µM; Sigma Aldrich, USA), MRS2179 (P_2_Y_1_ receptor blocker, F_c_ 100 µM; Sigma Aldrich, USA) or cangrelor (P_2_Y_12_ receptor blocker, F_c_ 100 nM; Sigma Aldrich, USA) before cells were activated with ADP (20 µM, 15 min, 37 °C). After lysis of red blood cells (BD FACS™ Lysing Solution, USA; 15 min, RT) samples were centrifuged (5 min, 450 g, RT) and the pellets washed in 2 ml PBS + Ca^2+^/Mg^2+^  + 0.1% BSA, before the samples were resuspended in 50 µl PBS + Ca^2+^/Mg^2+^  + 0.1% BSA and incubated with the scFv MAN-1 (F_c_ = 10 µg/ml, 15 min, 4 °C). Detection of MAN-1 binding was done with 1 μl of an Alexa Fluor 488 anti-His-Tag antibody (15 min, 4 °C; Qiagen, Germany); at the same time, 10 μl of an anti-CD14-PE antibody (Beckman Coulter, USA; 15 min, 4 °C) was added in order to identify the monocytes. Unbound antibodies were eliminated in a further washing step (5 min, 450 g, RT) and the pellets consisting of stained leukocytes were resuspended in 500 μl CellFix solution (BD Bioscience, USA). MAN-1 binding was quantified in FITC median of all CD14^+^ monocytes indirectly using the secondary Alexa Fluor 488 anti-His-Tag antibody. Percentage change from baseline MAN-1 binding was calculated in the following way: 100% x ((ADP-stimulated (± blockers) MAN-1 binding—PBS-stimulated MAN-1 binding) / PBS-stimulated MAN-1 binding).

Since clopidogrel is a prodrug which is activated in the liver after oral ingestion, it was not used in ex vivo experiments. Ticagrelor, another P_2_Y_12_ receptor blocker, is also intended for oral intake and was therefore also not used in experiments where P_2_Y_12_ receptor blockers were applied in solution. Instead, we performed experiments with cangrelor, which is a direct P_2_Y_12_ receptor blocker that is fully active in solution and is therefore applied intravenously in patients, or another experimental P_2_Y_12_ receptor blocker, 2MeSAMP.

To identify MPA by flow cytometry from citrated whole blood, samples were processed as described above but not stimulated. MPA were not stained with MAN-1. They were resuspended in 50 µl PBS + Ca^2+^/Mg^2+^  + 0.1% BSA and stained with 10 μl PE anti-CD14 and 10 μl FITC anti-CD41 or an appropriate FITC isotype-control antibody (both: Beckman Coulter, USA; 15 min, RT).

To identify MPA by flow cytometry from monocytes isolated by Ficoll density gradient, elutriation or cell sorting, cells (50 µl final volume per sample with a concentration of 500,000 cells/ml) were also stained with 10 μl PE anti-CD14 (except for sorted cells, which had already been stained) and 10 μl FITC anti-CD41 or a FITC isotype-control antibody (both: Beckman Coulter, USA; 15 min, RT).

MPA were defined as CD14^+^ monocytes that are positive for CD41, which is presented as the FITC median. Acquisition was stopped after 2000 monocytes were acquired. Acquisition and analysis were performed using a FACS Canto II Flow cytometer and FACS DIVA 8.0 software (Becton Dickinson, USA).

### Cell culture

THP-1 cells were cultured using 75 cm^2^ cell-culture flasks with RPMI 1640 medium (37 °C, 5% CO_2_). THP-1 cells (7 × 10^5^/ml) (ATCC, USA) were differentiated into THP-1 macrophages with PMA (F_c_ 200 ng/ml, up to 72 h, 37 °C, 5% CO_2_). Differentiation was visually confirmed by morphological change and adherence to the surface of the cell-culture flask.

### RT-PCR for P_2_Y_12_ receptor expression

RNA of 5 × 10^6^ THP-1 cells or macrophages was isolated with Trizol® (Invitrogen, USA) as recommended in the supplier’s manual. RNA concentration was determined using Nanodrop (ThermoFisher Scientific, USA) and samples were immediately stored at − 80 °C. RNA was transcribed into cDNA using Taqman® Reverse Transcription Reagents (Applied Biosystems, USA) as recommended by the manufacturer. RT-PCR was performed using standard protocols with the GoTaq® Green Master Mix (Promega, USA). CCAGAATCAACAGTTATCAGGTAACC was used as a forward primer and GTCAGTTAATATTTTTACTTAGCGCTTTGC as a reverse primer. All RT-PCR were repeated 3 times (3 independent biological replications). PCR products were run on a 1% agarose gel together with a DNA ladder, stained with ethidium bromide and quantified with Chemidoc™ (Biorad, USA).

### mRNA profiling of THP-1 monocytes and macrophages

To assess the expression of different ADP-induced genes in macrophages, THP-1 monocytes were differentiated into THP-1 macrophages as described above in the section “Cell culture”. THP-1 macrophages were then stimulated with ADP (20 µM, 24 h, 37 °C) and RNA was isolated. qRT-PCR using Fast SYBR® Green and commercially available primers (both: Applied Biosystems, USA) was used to determine the expression levels of inflammatory genes such as TNF-α, IL-1b, IL-8 and MMP-9. RPS18 was used as a housekeeping gene. Fold change of expression was defined in relation to the housekeeping gene as 2^−∆Ct^.

To assess whether the macrophage P_2_Y_12_ receptors have any functional impact on inflammatory gene expression, mRNA microarrays (Illumina, USA) were performed on RNA isolated from THP-1 monocytes, THP-1 macrophages treated with PBS and THP-1 macrophages that had been stimulated with the P_2_Y_12_ receptor agonist ADP (20 µM, 24 h, 37 °C). Total RNA isolation was the same as above from n = 3 biologically independent samples. For gene expression analysis, biotin-labelled cRNA was produced by means of a linear amplification kit (AMIL1791, Ambion, Austin, TX) using 250 ng quality-checked total RNA as input. Hybridisation to the Illumina BeadChip was for 16 h at 58 °C on a BeadChip Hyb Wheel using 1500 ng biotin-labelled cRNA as input. The chip was then washed, blocked and stained with Cy3-streptavidin (Amersham Bioscience, Piscataway, NJ, USA) according to the manufacturer’s protocols (Illumina, San Diego, CA, USA). iScan Control Software V1.6.20.7 (Illumina), together with the Illumina iSCAN platform (Illumina), was used for RNA quantification. Processing and analysis of the microarray data were performed with the Illumina Beadstudio 3.1.3.0 software as described previously^67,68^. Selection of differentially expressed genes was performed on the basis of reference to PBS-treated THP-1 macrophages or THP-1 monocytes as thresholds for fold changes plus statistical significance according to an Illumina custom model (*p* < 0.05) and fold change of greater than 2 or less than 0.5.

### Immunofluorescence microscopy of human carotid plaques

Human endarterectomy segments were frozen in Tissue Tek (Sakura Finetek, Germany) and stored at − 80 °C. Plaques were cut into 6 µm thick serial sections and immobilised on microscope slides. Tissue sections were rehydrated and blocked with 10% normal goat serum (Vector Laboratories, USA). After blocking, the sections were incubated with mouse anti-human CD68 primary antibody (Dako, USA) and rabbit polyclonal P_2_Y_12_ primary antibody (Cat: GTX54796, GeneTex, USA) at 4 °C overnight. As controls, samples were stained with mouse IgG1κ isotype (BioLegend, USA), rabbit IgG isotype (Vector Laboratories) and only the primary antibody. Sections were subsequently incubated with an Alexa Fluor 555–conjugated goat anti-mouse secondary antibody (Thermo Fisher, USA) and an Alexa Fluor 647–conjugated goat anti-rabbit secondary antibody (Thermo Fisher, USA) for 30 min at RT. Nuclei were stained with DAPI. All images were captured using a Nikon A1 Confocal Laser Microscope System and processed using Fiji software.

### Dynamic adhesion assays

Capillaries (Vitrotubes Rectangle Capillaries, 0.20 × 2.00 mm, Vitrocom, New Jersey, USA) were coated with ICAM-1 (F_c_ 40 pM, overnight, 4 °C), a main ligand of activated Mac-1 receptors^69^, and blocked with 1% BSA (1 h, RT). To obtain MPA, THP-1 cells were adjusted to 10^6^/ml and incubated with washed platelets which had been isolated as described previously^70,71^. As controls, THP-1 cells (± ADP stimulation) which had not been incubated with platelets were also run. After incubation, P_2_Y_12_ receptor blockers (2MeSAMP, F_c_ 100 µM; cangrelor F_c_ 2 µM; or active prasugrel metabolite; SiChem, Germany; F_c_ 0.25 µM) or PBS were added, followed by stimulation with ADP (20 µM) or PBS. THP-1 cells were perfused through the capillaries at 0.04 ml/min for 5 min using a syringe pump (PhD 2000, Harvard Apparatus). Adhesion of THP-1 cells to ICAM-1 was determined by counting attached THP-1 monocytes/cm^2^ after 5 min perfusion using an IX81 Olympus microscope.

### Transmigration assay

In order to investigate whether ADP might be a chemoattractant targeting P_2_Y_12_-expressing THP-1 macrophages, a transmigration assay was conducted. Therefore, 4 × 10^5^ THP-1 monocytes were differentiated into macrophages in the inserts of a transwell assay. Differentiated macrophages were either pre-incubated with P_2_Y_12_ receptor blockers (2MeSAMP, F_c_ 100 µM; cangrelor, F_c_ 1 µM; 30 min, 37 °C) or PBS before different concentrations of ADP (F_c_ 1 µM, 2 µM, 5 µM) were placed in the cell-culture flask bottom wells and macrophages were seeded into the cell-culture well inserts (8 µm pore size; BD, USA). MCP-1 (10 ng/µl) served as a positive control. After 4 h, the numbers of transmigrated THP-1 macrophages were quantified with a Neubauer chamber (LoLaboroptik, UK).

### Statistics

The means of 2 continuous variables were compared using a two-tailed Student’s t-test, as indicated. Analysis of 3 or more variables was performed using one-way ANOVA and Tukey’s post-hoc test. Statistical analysis of the RNA microarray experiments was performed as described above. Data are presented as mean ± SEM. A *p*-value < 0.05 was considered statistically significant. If not stated otherwise, all experiments were repeated at least 3 times. Statistical analysis was performed using GraphPad Prism 8 (GraphPad Software, USA).

## Supplementary Information

Supplementary Figures.

Supplementary Table S1.

Supplementary Table S2.

## Data Availability

All data are available from the authors upon reasonable request.
