# Supplementary material for: P2Y12 receptor blockers are anti-inflammatory drugs inhibiting both circulating monocytes and macrophages including THP-1 cells
Source: Sci Rep. 2021 Aug 31;11:17459. doi: 10.1038/s41598-021-95710-3 (PMC8408182; doi:10.1038/s41598-021-95710-3)
Supplement: Supplementary file 1 — Supplementary Figures. [file 41598_2021_95710_MOESM1_ESM.pdf]

P_2Y_{12} receptor blockers are anti-inflammatory drugs inhibiting both circulating monocytes and macrophages including THP-1 cells

Patrick M. Siegel, M. D.[1,2]; Laura Sander, M. D.[2]; Alba Fricke, M. D.[2]; Johannes Stamm, M. D.[2]; Xiaowei Wang, Ph. D.[2,3,4]; Prerna Sharma[2]; Nicole Bassler[2]; Ya-Lan Ying, Ph. D.[2], Christoph B. Olivier, M. D.[1]; Steffen U. Eisenhardt, M. D.[2,5]; Christoph Bode, M. D.[1]; Ingo Ahrens, M. D.[1,2,6]; Philipp Diehl, M. D., Ph. D.[1,2,3#] & Karlheinz Peter, M. D., Ph. D.[1,2,3,4#]

[1] Department of Cardiology and Angiology I, University Heart Center Freiburg – Bad Krozingen, Faculty of Medicine, University of Freiburg, Freiburg, Germany

[2] Atherothrombosis and Vascular Biology Laboratory, Baker Heart and Diabetes Institute, Melbourne, Australia

[3] Department of Medicine, Central Clinical School, Monash University, Melbourne, Australia

[4] Baker Department of Cardiometabolic Health, University of Melbourne, Melbourne, Australia

[5] Department of Plastic and Hand Surgery, Faculty of Medicine, University of Freiburg, Freiburg, Germany

[6] Department of Cardiology and Medical Intensive Care, Augustinerinnen Hospital, Cologne, Germany

[#]equally contributing senior authors

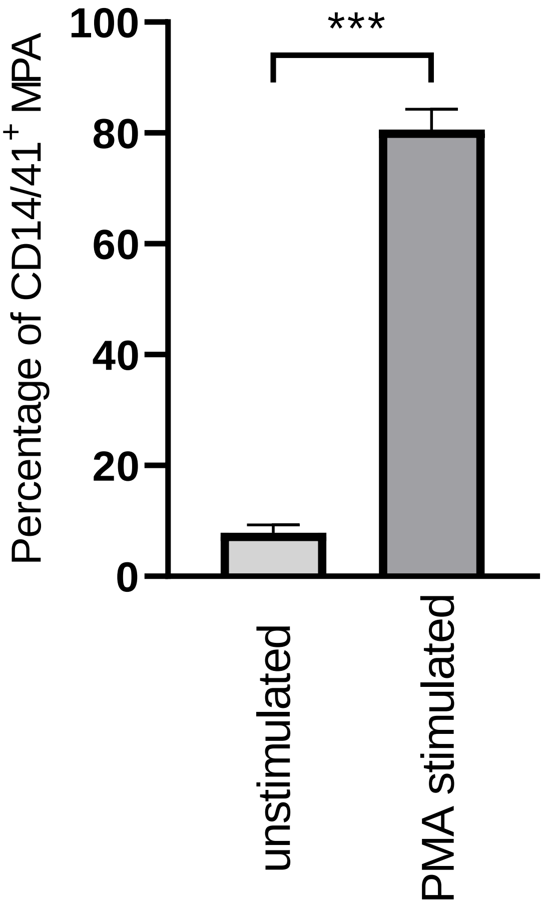

Supplementary Figure S1. CD14/41$^+$ monocyte-platelet aggregates (MPA) in lysed whole blood from healthy volunteers in percentage of CD14$^+$ monocytes. Blood sampling, preparation and staining were performed as described in the Methods section. Blood was stimulated with phorbol 12-myristate 13-acetate (200ng/ml) for 15 min to induce MPA formation. The percentage of MPA in lysed whole blood was approximately 7.8%. Data are presented as mean±SEM. n=9, ***$p<0.001$, p-values were calculated by an unpaired t-test.

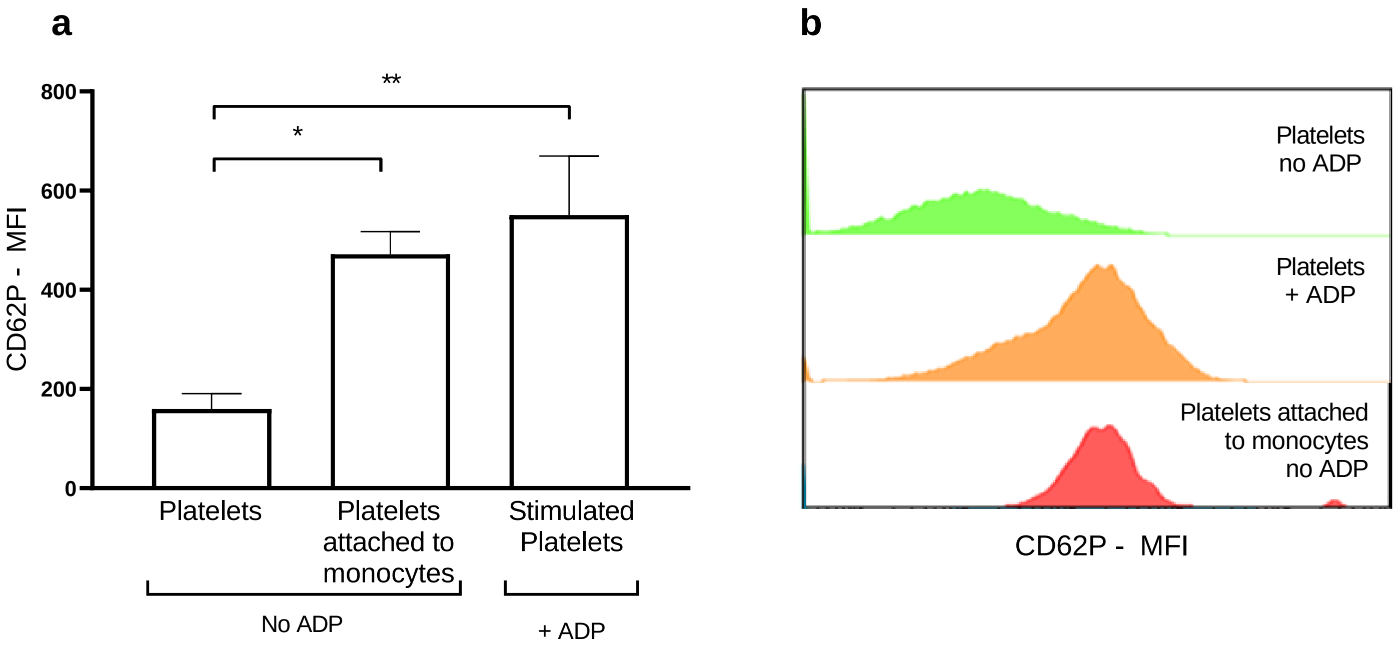

Supplementary Figure S2. Platelet CD62P expression in lysed whole blood from healthy volunteers. (**a**) Bar graphs showing the mean CD62P MFI values in the different treatment groups. (**b**) Representative histograms of the different treatment groups. Blood preparation and flow cytometry was very similar to the protocol described in the Methods section for the MPA analysis from lysed whole blood. Platelets were identified by their CD41 expression and stained with an anti-CD62P antibody. CD62P expression was quantified by mean fluorescence intensity on unstimulated platelets, unstimulated platelets attached to monocytes and ADP (20 µM)-stimulated platelets. Data are presented as mean±SEM. n=5, *$p<0.05$, **$p<0.01$, p-values were calculated by one-way ANOVA with a Tukey's multiple comparison post-test.

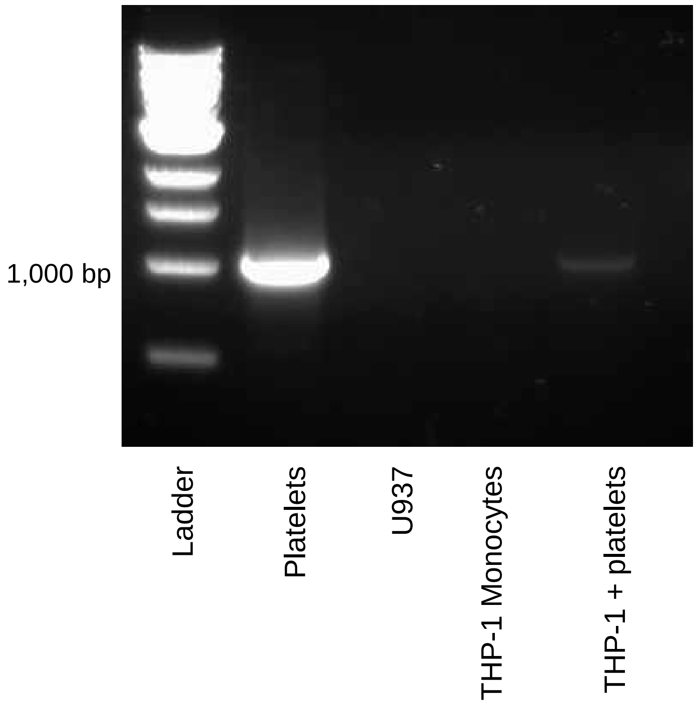

Supplementary Figure S3.
Uncropped 1% agarose gel of Figure 4a showing RT-PCR results using primers specific for the P_2Y_{12} receptor as described in the Methods section.

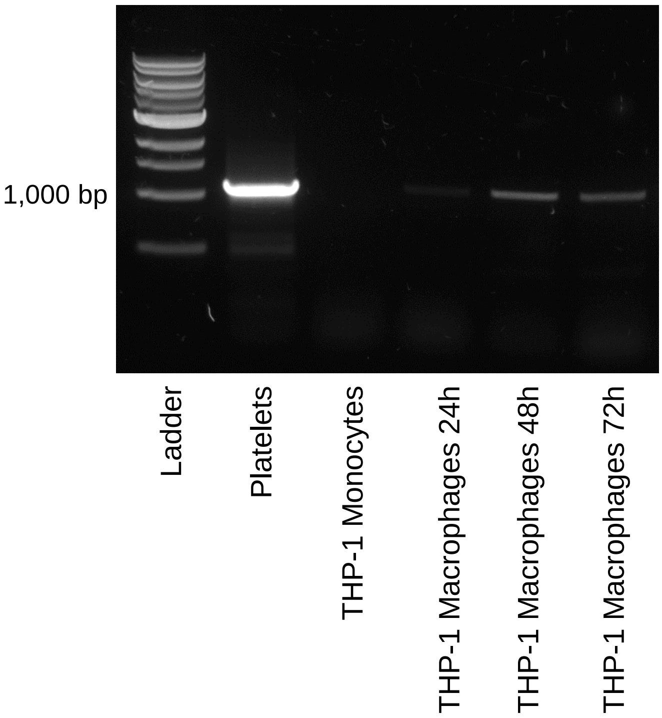

Supplementary Figure S4.
Uncropped 1% agarose gel of Figure 4b showing RT-PCR results using primers specific for the P_2Y_{12} receptor as described in the Methods section.
