# Supplementary material for: P2Y12 receptor blockers are anti-inflammatory drugs inhibiting both circulating monocytes and macrophages including THP-1 cells
Source: Sci Rep. 2021 Aug 31;11:17459. doi: 10.1038/s41598-021-95710-3 (PMC8408182; doi:10.1038/s41598-021-95710-3)
Supplement: Supplementary file 2 — Supplementary Table S1. [file 41598_2021_95710_MOESM2_ESM.pdf]

P_2Y_{12} receptor blockers are anti-inflammatory drugs inhibiting both circulating monocytes and macrophages including THP-1 cells

Patrick M. Siegel, M. D.[1,2]; Laura Sander, M. D.[2]; Alba Fricke, M. D.[2]; Johannes Stamm, M. D.[2]; Xiaowei Wang, Ph. D.[2,3,4]; Prerna Sharma[2]; Nicole Bassler[2]; Ya-Lan Ying, Ph. D.[2], Christoph B. Olivier, M. D.[1]; Steffen U. Eisenhardt, M. D.[2,5]; Christoph Bode, M. D.[1]; Ingo Ahrens, M. D.[1,2,6]; Philipp Diehl, M. D., Ph. D.[1,2,3#] & Karlheinz Peter, M. D., Ph. D.[1,2,3,4#]

[1] Department of Cardiology and Angiology I, University Heart Center Freiburg – Bad Krozingen, Faculty of Medicine, University of Freiburg, Freiburg, Germany

[2] Atherothrombosis and Vascular Biology Laboratory, Baker Heart and Diabetes Institute, Melbourne, Australia

[3] Department of Medicine, Central Clinical School, Monash University, Melbourne, Australia

[4] Baker Department of Cardiometabolic Health, University of Melbourne, Melbourne, Australia

[5] Department of Plastic and Hand Surgery, Faculty of Medicine, University of Freiburg, Freiburg, Germany

[6] Department of Cardiology and Medical Intensive Care, Augustinerinnen Hospital, Cologne, Germany

[#]equally contributing senior authors

P VALUE	GENE	SUGGESTED FUNCTION	REFERENCE
0.002	HIPK2	• Mediating apoptosis	[1]
0.028	CCL3L1	• Increased in coronary artery lesion<br>• pro-inflammatory	[2]
0.01	TGFB3	• cell proliferation + migration, vessel stiffness<br>• increased in lipid-rich intimal lesions	[3]
0.008	RGS16	• induces cardiac failure<br>• induces lymphocyte migration	[4, 5]
0.038	CCL1	• induces inflammation by WBC recruitment	[6]
0.042	ADAMDEC1	• increased in unstable plaque vs. stable plaque	[7]
0.003	ZC3H12A	• encodes for MCPIP1	[8]
0.029	TNF	• induces apoptosis	[9]
0.033	SDC4	• enhanced lipid uptake	[10]
0.027	MTE	• present in carotid plaque	[11]
0.032	EBI3	• expressed in plaque<br>• pro-atherothrombotic	[12, 13]
7.18E-04	VEGFA	• neo-angiogenesis<br>• associated with. stroke	[14]
0.017	CXCR5	• enhances atherosclerosis	[15]
0.007	UCN2	• UCN1 induces inflammation in endothelial cells	[16]
0.014	TRAF1	• increased in atherosclerosis	[17]
0.011	ICAM5	• leukocyte recruitment via β2 integrins	[18]
0.023	SRC	• attenuates atherosclerosis	[19]
0.03	CXCL1	• induces monocyte adhesion & atherosclerosis	[20]
0.03	S100A8	• increases atherogenesis	[21]
9.88E-04	THBS4	• encodes for TSB-4, which is abundant in atherosclerotic lesions	[22]
0.027	CCL3	• Increased in cardiac ischemia	[23]

Supplementary Table S1. Examples of upregulated genes in macrophages after ADP stimulation. Several genes, which are involved in vascular inflammation and pathogenesis of atherosclerosis, were upregulated in ADP-stimulated THP-1 macrophages vs. THP-1 macrophages that were incubated with PBS (control). n= 3 independent biological samples.

References

1 McDonough, H. *et al.* Stress-dependent Daxx-CHIP interaction suppresses the p53 apoptotic program. *J Biol Chem* **284**, 20649-20659. doi:M109.011767 [pii] 10.1074/jbc.M109.011767 (2009).

2 Mamtani, M. *et al.* Association of CCR2-CCR5 haplotypes and CCL3L1 copy number with Kawasaki Disease, coronary artery lesions, and IVIG responses in Japanese children. *PLoS One* **5**, e11458. doi:10.1371/journal.pone.0011458 (2010).

3 Bobik, A. *et al.* Distinct patterns of transforming growth factor-beta isoform and receptor expression in human atherosclerotic lesions. Colocalization implicates TGF-beta in fibrofatty lesion development. *Circulation* **99**, 2883-2891 (1999).

4 Lippert, E. *et al.* Role of regulator of G protein signaling 16 in inflammation-induced T lymphocyte migration and activation. *J Immunol* **171**, 1542-1555 (2003).

5 Patten, M. *et al.* Endotoxin induces desensitization of cardiac endothelin-1 receptor signaling by increased expression of RGS4 and RGS16. *Cardiovasc Res* **53**, 156-164. doi:S0008636301004436 [pii] (2002).

6 Hoshino, A. *et al.* Inhibition of CCL1-CCR8 interaction prevents aggregation of macrophages and development of peritoneal adhesions. *J Immunol* **178**, 5296-5304. doi:178/8/5296 [pii] (2007).

7 Papaspyridonos, M. *et al.* Novel candidate genes in unstable areas of human atherosclerotic plaques. *Arterioscler Thromb Vasc Biol* **26**, 1837-1844. doi:01.ATV.0000229695.68416.76 [pii] 10.1161/01.ATV.0000229695.68416.76 (2006).

8 Cifuentes, R. A., Cruz-Tapias, P., Rojas-Villarraga, A. & Anaya, J. M. ZC3H12A (MCPIP1): molecular characteristics and clinical implications. *Clin Chim Acta* **411**, 1862-1868. doi:S0009-8981(10)00530-9 [pii] 10.1016/j.cca.2010.08.033 (2010).

9 Wang, S. & El-Deiry, W. S. TRAIL and apoptosis induction by TNF-family death receptors. *Oncogene* **22**, 8628-8633 (2003).

10 Boyanovsky, B. B., Shridas, P., Simons, M., van der Westhuyzen, D. R. & Webb, N. R. Syndecan-4 mediates macrophage uptake of group V secretory phospholipase A2-modified LDL. *J Lipid Res* **50**, 641-650. doi:M800450-JLR200 [pii] 10.1194/jlr.M800450-JLR200 (2009).

11 Daskalopoulou, S. S. *et al.* Metallothionein expression in the high-risk carotid atherosclerotic plaque. *Curr Med Res Opin* **23**, 659-670 (2007).

12 Kempe, S. *et al.* Epstein-barr virus-induced gene-3 is expressed in human atheroma plaques. *Am J Pathol* **175**, 440-447 (2009).

13 Ait-Oufella, H., Taleb, S., Mallat, Z. & Tedgui, A. Recent advances on the role of cytokines in atherosclerosis. *Arterioscler Thromb Vasc Biol* **31**, 969-979.

14 Nagy, J. A., Dvorak, A. M. & Dvorak, H. F. VEGF-A(164/165) and PlGF: roles in angiogenesis and arteriogenesis. *Trends Cardiovasc Med* **13**, 169-175. doi:S1050173803000562 [pii] (2003).

15 Daissormont, I. *et al.* The CXCL13-CXCR5 axis contributes to atherosclerosis development in LDLr-/- mice by regulating myeloid development. *The Journal of Immunology,* **2011**, 149.141 (2011).

16 Zhang, R. *et al.* Urocortin induced expression of COX-2 and ICAM-1 via corticotrophin-releasing factor type 2 receptor in rat aortic endothelial cells. *Br J Pharmacol* **158**, 819-829 (2009).

17 Missiou, A. *et al.* Tumor necrosis factor receptor-associated factor 1 (TRAF1) deficiency attenuates atherosclerosis in mice by impairing monocyte recruitment to the vessel wall. *Circulation* **121**, 2033-2044. doi:CIRCULATIONAHA.109.895037 [pii] 10.1161/CIRCULATIONAHA.109.895037 (2010).

18 Tian, L. *et al.* Intercellular adhesion molecule-5 induces dendritic outgrowth by homophilic adhesion. *J Cell Biol* **150**, 243-252 (2000).

19 Silverstein, R. L., Li, W., Park, Y. M. & Rahaman, S. O. Mechanisms of cell signaling by the scavenger receptor CD36: implications in atherosclerosis and thrombosis. *Trans Am Clin Climatol Assoc* **121**, 206-220 (2010).

20 Zhou, Z. *et al.* Lipoprotein-derived lysophosphatidic acid promotes atherosclerosis by releasing CXCL1 from the endothelium. *Cell Metab* **13**, 592-600.

21 McCormick, M. M. *et al.* S100A8 and S100A9 in human arterial wall. Implications for atherogenesis. *J Biol Chem* **280**, 41521-41529 (2005).

22 Frolova, E. G. *et al.* Thrombospondin-4 regulates vascular inflammation and atherogenesis. *Circ Res* **107**, 1313-1325.

23 de Jager, S. C. *et al.* CCL3 (MIP-1 alpha) levels are elevated during acute coronary syndromes and show strong prognostic power for future ischemic events. *J Mol Cell Cardiol* **45**, 446-452. doi:S0022-2828(08)00499-9 [pii] 10.1016/j.yjmcc.2008.06.003 (2008).
